# Modified Perfluorocarbon Tracer Method for Measuring Effective Multizone Air Exchange Rates

**DOI:** 10.3390/ijerph7093348

**Published:** 2010-08-27

**Authors:** Naohide Shinohara, Toshiyuki Kataoka, Koichi Takamine, Michio Butsugan, Hirokazu Nishijima, Masashi Gamo

**Affiliations:** 1 Research Institute of Science for Safety and Sustainability (RISS), National Institute of Advanced Industrial Science and Technology (AIST), 16-1 Onogawa, Tsukuba, Ibaraki 305-8569, Japan; E-Mail: masashi-gamo@aist.go.jp; 2 The Chemicals Evaluation and Research Institute, Japan (CERI), 1600 Shimotakano, Sugito-machi, Kitakatsushika-gun, Saitama 345-0043, Japan; E-Mails: takamine-koichi@ceri.jp (T.K.); kataoka-toshiyuki@ceri.jp (K.T.); 3 Hitachi Chemical Co., Ltd., 13-1, Higashi-cho 4-chome, Hitachi-shi, Ibaraki 317-8555, Japan; E-Mail: m-butsugan@hitachi-chem.co.jp; 4 Sigma-Aldrich Japan K.K., 2-2-24, Higashi-Shinagawa, Shinagawa-ku, Tokyo 140-0002, Japan; E-Mail: hnishijima@sial.com

**Keywords:** air exchange rate, 24-h average, PFT method, multizone, solvent extraction

## Abstract

A modified procedure was developed for the measurement of the effective air exchange rate, which represents the relationship between the pollutants emitted from indoor sources and the residents’ level of exposure, by placing the dosers of tracer gas at locations that resemble indoor emission sources. To measure the 24-h-average effective air exchange rates in future surveys based on this procedure, a low-cost, easy-to-use perfluorocarbon tracer (PFT) doser with a stable dosing rate was developed by using double glass vials, a needle, a polyethylene-sintered filter, and a diffusion tube. Carbon molecular sieve cartridges and carbon disulfide (CS_2_) were used for passive sampling and extraction of the tracer gas, respectively. Recovery efficiencies, sampling rates, and lower detection limits for 24-h sampling of hexafluorobenzene, octafluorotoluene, and perfluoroallylbenzene were 40% ± 3%, 72% ± 5%, and 84% ± 6%; 10.5 ± 1.1, 14.4 ± 1.4, and 12.2 ± 0.49 mL min^−1^; and 0.20, 0.17, and 0.26 μg m^−3^, respectively.

## 1. Introduction

Since the 1990s, Japanese buildings have been designed to be more tightly sealed off from the external atmosphere. This has exacerbated indoor air pollution, leading to problems such as sick building syndrome and multiple chemical sensitivities in some houses in Japan [1]. One of the most effective ways to reduce the concentration of indoor air pollutants is to increase ventilation [2,3].

To study the degree of the problem, researchers must conduct surveys of the air exchange rates in many houses. For such studies, it would be quite desirable to have a low-cost measurement device that can be operated by nonexperts such as the residents themselves. Furthermore, useful data would be obtained if the device could separately measure the rates of exfiltration and infiltration between the indoor and outdoor environments (*i.e.*, the indoor-outdoor air exchange rates) and also those between the individual rooms (inter-room air exchange rates) over a period of time while the occupants continue with their usual life.

To this end, several methods have been proposed: the carbon dioxide (CO_2_) concentration decay method [4], CO_2_ or sulfur hexafluoride (SF_6_) constant concentration method [5], SF_6_ tracer gas method [6], and perfluorocarbon tracer (PFT) method [7–9] among others. The CO_2_ and SF_6_-based methods, however, are not promising. The CO_2_ concentration decay method is not applicable for extended measurements while the occupants continue with their usual life, but is instead suitable for rapid measurement of the air exchange rates through an entire house or room. Although the SF_6_ constant concentration method is the most precise [10], it requires expensive instruments and technical handling. In addition, methods relying on a single tracer gas, either SF_6_ or CO_2_, cannot measure the exchange rates across multiple rooms: the inter-room air exchange rate cannot be separated from the total indoor-outdoor air exchange rate. By contrast, with the PFT method, multiple PFTs can be used in different rooms to obtain the individual inter-room air exchange rates.

This PFT method was developed by Dietz *et al.* [7] and applied to the survey of air exchange rates in the residential houses [3]. The tracer gases, PFTs, are passively emitted and sampled using diffusion source (doser) and diffusion samplers. This doser is costly and does not allow for the convenient exchange or replenishment of the tracer gas. In addition, the dosing rates of the tracer gas source decrease with time.

The drawback of these methods is that a perfect mixing state is presumed, which could introduce erroneous data when the indoor convection flow is lopsided or regional ventilation is used [11]. Dodson *et al.* recommended that multiple PFT source and sampling points are placed within the structure to avoid the possible systematic errors because the sampling point is possible to be upwind or downwind of PFT source when a single source is used [12]. If the dosers of the tracer gas are placed at multiple points in a room that resemble indoor emission sources, such as the four corners of the room, the effective air exchange rate calculated from the average indoor concentration at center of the room and total amount of tracer gas emitted is more appropriate for an assessment of the contribution of emissions from indoor sources to the occupants’ level of exposure regardless of the wind directions. For this purpose, we developed a low-cost and easy-to-use doser with stable dosing rates because a large number of dosers of tracer gas are required in a large-scale survey of air exchange rates. Note that hereinafter, the “air exchange rates” refer to the “effective air exchange rates”, unless otherwise stated.

With this background, the objectives of this study were as follows: (1) to develop a new PFT doser that is inexpensive, can be operated by nonexpert volunteer residents, has a stable dosing rate, and whose tracer gas can be easily refilled; and (2) to test and discuss the applicability of a modified PFT method employing the developed doser for a week in a survey of the 24-h-average multiroom air exchange rates in a Japanese house.

## 2. Materials and Methods

### 2.1. Modified PFT Method

In previous studies, PFT dosers were often placed at one to three points in a room, such as near the air inlet or on the furniture, according to the predicted air pathways [8,13]. Indoor pollutants, however, are not emitted from the air inlet but rather from the surfaces of sources such as the walls, flooring, and furniture; these pollutants are also diluted by fresh air entering from outdoors. The modified method proposed in the present study can be used to determine the effective air exchange rates after taking into account the emission of pollutants from various sources; this can be achieved by placing the dosers of tracer gas at indoor locations that resemble emission sources. For example, placing the dosers at the four corners of a room can create flows that are similar to those generated during the emission and migration of actual pollutants from building materials. In addition, the systematic error due to the wind direction is possible to be avoided by this placing way [12].

In this study, four PFT dosers dispensing the same kind of PFT were placed at the four corners of a room at a height of 200 cm. The same was done in each of the other rooms but with other kinds of PFTs—a different kind in each room. More than 12 h after the placing the dosers, the air was passively sampled with a carbon molecular sieve cartridge (VOC-SD; Sigma-Aldrich Japan K.K., Japan), which can be used to simultaneously sample VOCs. The cartridges were hung from the ceiling at the center of each room at a height of 1.8 m for 24 h.

**Figure 1.**
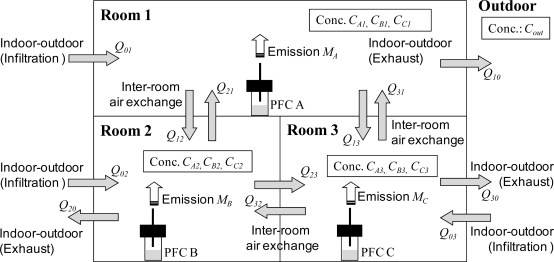
Indoor-outdoor and inter-room air and PFT flows.

Consider a three-room house as shown in Figure 1. We assume steady-state conditions, perfect mixing in each room, and zero outdoor PFT concentration. Then the air exchange rates (three indoor-outdoor air infiltration rates, three indoor-outdoor air exhaust rates, and six inter-room air exchange rates) can be represented by nine mass-balance equations for the amount of PFTs and three mass-balance equations for the volume of air (Figure 1) [8].

In this study, the air flow rates were optimized by quasi-Newton’s method with the constraint *Q* > 0 to minimize the square sum of the differences between the left and right sides of the 12 mass-balance equations by using the solver function in Microsoft Excel 2003:

(1) $$\begin{array}{l} C_{A2}\times Q_{21}+C_{A3}\times Q_{31}-C_{A1}\times (Q_{10}+Q_{12}+Q_{13})=-M_{A}\\ C_{A1}\times Q_{12}+C_{A3}\times Q_{32}-C_{A2}\times (Q_{21}+Q_{23}+Q_{20})=0\\ C_{A1}\times Q_{13}+C_{A2}\times Q_{23}-C_{A3}\times (Q_{31}+Q_{32}+Q_{30})=0\\ C_{B1}\times Q_{12}+C_{B3}\times Q_{32}-C_{B2}\times (Q_{20}+Q_{21}+Q_{23})=-M_{B}\\ C_{B2}\times Q_{21}+C_{B3}\times Q_{31}-C_{B1}\times (Q_{12}+Q_{13}+Q_{10})=0\\ C_{B1}\times Q_{13}+C_{B2}\times Q_{23}-C_{B3}\times (Q_{31}+Q_{32}+Q_{30})=0\\ C_{C1}\times Q_{13}+C_{C2}\times Q_{23}-C_{C3}\times (Q_{30}+Q_{31}+Q_{32})=-M_{C}\\ C_{C2}\times Q_{21}+C_{C3}\times Q_{31}-C_{C1}\times (Q_{12}+Q_{13}+Q_{10})=0\\ C_{C1}\times Q_{12}+C_{C3}\times Q_{32}-C_{C2}\times (Q_{21}+Q_{23}+Q_{20})=0\\ Q_{01}-Q_{10}-Q_{12}+Q_{21}-Q_{13}+Q_{13}=0\\ Q_{02}-Q_{20}-Q_{21}+Q_{12}-Q_{23}+Q_{32}=0\\ Q_{03}-Q_{30}-Q_{31}-Q_{13}-Q_{32}+Q_{23}=0\\ \left(Q>0\right) \end{array}$$

### 2.2. Candidate Tracer Gases

Five candidate PFTs were tested: perfluoromethylcyclohexane (PMCH; Alfa Aesar, USA), perfluoro-1,2-dimethylcyclohexane (PDCH; Fluorochem Ltd., USA), hexafluorobenzene (HxFBz; Wako Pure Chemical Industries, Ltd., Japan), octafluorotoluene (OFT; Wako Pure Chemical Industries, Ltd., Japan), and perfluoroallylbenzene (PFABz; Tokyo Chemical Industry Co., Ltd., Japan).

### 2.3. Analysis of Extracted PFTs

The PFTs captured in the cartridges were extracted with carbon disulfide (CS_2_; Wako Pure Chemical Industries, Ltd., Japan) or hexane (Sigma-Aldrich, Inc., USA) by ultrasonication for 5 min. The extracted PFTs were analyzed on a gas chromatograph-mass spectrograph (GC-MS; HP6890-5973; Hewlett-Packard Co. Ltd., USA). Toluene-d_8_ was used as an internal standard. The analytical conditions are listed in Table 1. Quantitative analysis was conducted in SIM mode and the monitored ions were as follows: HxFBz: 186, 117; OFT: 217, 236; PFABz: 217, 298; PMCH: 69, 131; PDCH: 69, 131. Calibration curves were linear between 0.01 and 100 μg·mL^−1^ except for the solution of PDCH in CS_2_.

### 2.4. Detection Limit

The lower limit of detection (LOD) and lower limit of quantification (LOQ) of the analysis were defined as 3σ and 10σ of the absolute amount of the analyte (*N* = 5). In addition, detection and quantification limits for 24-h sampling with the passive sampler were calculated on the basis of the analytical LOD, LOQ, recovery efficiencies, and sampling rates.

### 2.5. Recovery Efficiency

To select the suitable PFTs and solvent, the recovery efficiencies of absorbed PFTs from the VOC-SD cartridges were evaluated. Aliquots with 10 μL of a methanol solution of PFTs (1.0 μg per aliquot) were used to spike the adsorbent (carbon molecular sieve) placed in a vessel with a microsyringe (*N* = 6). The vessels were covered with tight-fitting caps for 60 min. Then, the adsorbed PFTs were extracted with CS_2_ or hexane and analyzed with the GC-MS.

### 2.6. Sampling Rate

**Figure 2.**
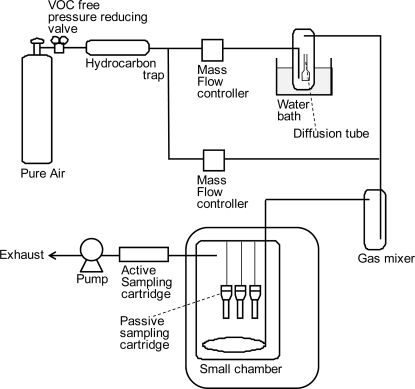
System for measurement of PFT sampling rate.

To obtain the sampling rates of PFTs for the VOC-SD, the passive sampling cartridges were left in a thermostatic chamber in which the PFT gases were passed through at stable concentrations for 24 h (*N* = 3; Figure 2). The sampling rates were calculated by comparing the amounts of PFTs adsorbed by the cartridge (passive sampling) to the in-chamber concentration measured by active sampling. Although the surrounding air flows in an experimental chamber and actual residential environments are likely to be different, the wind speed has little effect on the sampling rate of PFC because the diffusion resistance of the sampling cartridge is much larger than that of the boundary layer on the surface of the tubes [14].

### 2.7. Doser

**Figure 3.**
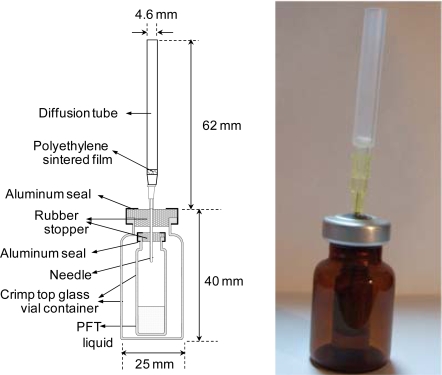
Photograph and schematic diagram of developed PFT doser. To start dosing, the doser has only to be pricked by a needle with a diffusion tube.

Figure 3 presents a photograph and schematic diagram of developed doser. To prevent leaks during transportation between the laboratory and measurement site, the device consists of two crimp-top glass vials with aluminum crimp seals and a rubber stopper. A needle (internal diameter: 0.61 mm) and polyethylene sintered filter maintain the dosing rate. The PFT liquid in the inner vial evaporates; diffuses through the needle, polyethylene sintered filter, and diffusion tube; and is emitted into the air. Due to the diffusion resistance, the indoor air flow is considered to have negligible effect on the dosing rate. To start dosing, the septa on both vials should be pierced with the needle. Expensive instruments such as a gas bottle, gas monitor, computer, and solenoid valve are not required for the method used to measure the air exchange rates in the present study. The PFT doser developed in this study consists of commercially available low-price glass vials and a needle. The doser also consists of a newly developed filter and a diffusion tube, which can be manufactured at low costs. An additional sampling cartridge is not required to be used because PFTs can be sampled in the same cartridge used for VOC sampling. One of the major advantages of this doser is that a constant dosing rate can be maintained despite disturbances during handling and transportation; another advantage is that this doser can be refilled with other tracer gases.

The dosing rate of the PFT doser was measured against time to confirm the stability. The decrease in weight of the doser was considered to be equal to the weight of PFT emitted. The dosing rates was measured by gravimetry every 48 h for 10 days in a thermostatic chamber (*N* = 10) at 5, 15, 25, 35, and 45 °C. To check leakage, the dosing rates from dosers with a no-hole needle were also measured over a period of 2 months.

### 2.8. Comparison with CO_2_ Concentration Decay Method

To validate the developed PFT method, the results were compared with those of the CO_2_ concentration decay method for a single room with a floor area of 17 m^2^ and volume of 46 m^3^. The inlet flow was stabilized with an inverter controller and the room air was not mixed to match realistic conditions. PFT (HxFBz, OFT, and PFABz) dosers were placed at each of the four corners of the room at a height of 200 cm for more than 12 h. Following this, passive sampling cartridges were placed at three central points at 1-m intervals and a height of 180 cm for 8 h. The CO_2_ concentration decay method was carried out in conformity with the JIS standard JIS A1406. CO_2_ gas was released from a gas bomb room until the indoor concentration reached 2000 ppm. Then the CO_2_ concentration was measured for 8 h with a Telaire 7001D (General Electric Company, USA). During the measurements, no one remained in the room.

### 2.9. Field Study

**Figure 4.**
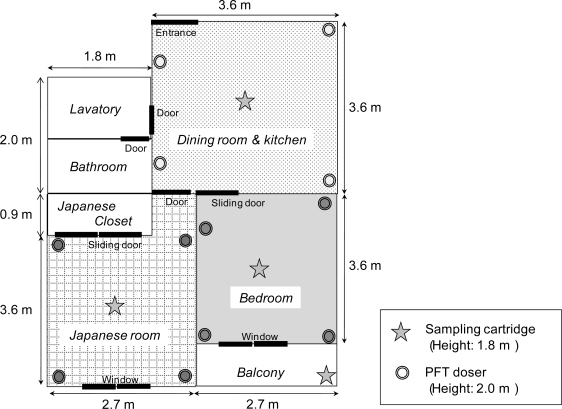
Layout of apartment in which measurements were taken, showing points where dosers and sampling cartridges were placed.

The air exchange rates were measured everyday for six days during summer in three rooms of a residential apartment in the Tokyo metropolitan area, Japan. Two persons went about their daily life during the tests. The residence is on the third floor of a five-year-old, four-story reinforced concrete building and has three rooms: dining room & kitchen, 13.2 m^2^ × 2.2 m; Japanese room, 10.7 m^2^ × 2.2 m; and bedroom, 9.9 m^2^ × 2.2 m (Figure 4). The volumes of the Japanese closet, bathroom and lavatory spaces were not included in the calculations of the air exchange rates because these spaces are not considered to be residential space. Because the frequency of opening and closing the Japanese closet was low (less than once a day) and the air flow due to the pressure difference between the room and closet seldom occurred, the closet space can be ignored in the calculations. If the volumes of the bathroom and lavatory spaces were included in the volume of the dining room & kitchen, the air exchange rates of the room could reduce by 30%.

## 3. Results and Discussion

### 3.1. Detection Limits, Recovery Efficiencies, and Sampling Rates

The results of the tests are listed in Table 2. The mean recovery efficiencies and their standard deviations (SDs) from the 10-μg spiked cartridges extracted with CS_2_ were 40% ± 3%, 72% ± 5%, 84% ± 6%, 2.7% ± 0.6%, and 24% ± 2% for HxFBz, OFT, PFABz, PMCH, and PDCH, respectively. The values obtained with hexane were <0.29%, 18% ± 2%, 54% ± 18%, and 84% ± 21% for HxFBz, OFT, PMCH, and PDCH, respectively. The precision was better for CS_2_, regardless of the recovery efficiency. Hence, CS_2_ was selected as the extraction liquid and HxFBz, OFT, and PFABz were selected as the tracer gases for three-zone air exchange measurement. The sampling rates of these three PFTs were 10.5 ± 1.1, 14.4 ± 1.4, and 12.2 ± 0.49 mL min^−1^. The detection and quantification limits for 24-h sampling calculated using the analytical LOD and LOQ and the sampling rates were 0.20, 0.17, and 0.26 μg m^−3^ and 0.66, 0.58, and 0.85 μg m^−3^ for HxFBz, OFT, and PFABz, respectively.

### 3.2. Dosing Rate

**Figure 5.**
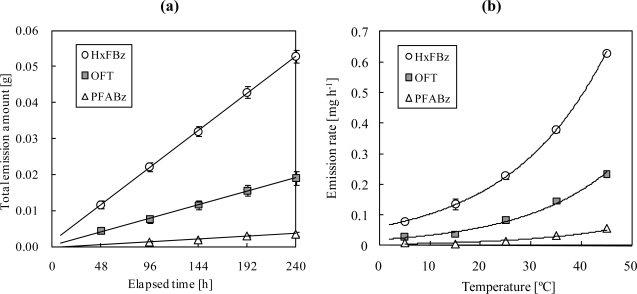
**(a)** Total dose of PFT against elapsed time (25 °C). **(b)** Temperature dependency of average dosing rate.

The total amount of PFTs released from the doser was linearly related to the elapsed time for 240 h (Figure 5(a)). The dosing rates for HxFBz, OFT, and PFABz were calculated as 0.23 ± 0.009, 0.082 ± 0.006, and 0.015 ± 0.002 mg h^−1^, respectively, at 25 °C, according to the slope of the correlation lines. The variance of the dosing rate was low for HxFBz and OFT among the 10 dosers (relative standard deviation (RSD) (= (SD/Mean) × 100): 2.6%–13% and 0.86%–13%, respectively). Although the variance for PFABz was low at 15–45 °C among the 10 dosers (RSD: 11%–16%), the dosing rate varied at 5 °C (RSD: 90%). In addition, the dosing rates exponentially increased with temperature (Figure 5(b)). Thus, to obtain more accurate air dosing rates, especially in winter, weight measurement of dosers before and after measurement of the air exchange rates is desirable. The PFT dose emitted from the device with the no-hole needle was 0.2 mg at 2 months, which is negligible.

### 3.3. Comparison with CO_2_ Concentration Decay Method

The air exchange rates measured by the PFT method did not differ at three points in the room (RSD < 10%). The exchange rates measured using HxFBz and OFT (1.0 ± 0.02 h^−1^, 0.95 ± 0.03 h^−1^) are in agreement with the results obtained by the CO_2_ concentration decay method (0.96 h^−1^). However, the result obtained with PFABz (1.4 ± 0.12 h^−1^) was 40% higher than that obtained by the CO_2_ concentration decay method. This could be due to analytical and gravimetric error: the amount of PFABz collected in the sampling cartridge was close to the LOQ (16–20 ng per cartridge), and the total dose of PFABz during an 8-h period was small (1.6 mg) owing to the relatively low vapor pressure of PFABz. To reduce the gravimetric error, it is recommended that a longer dosing duration be used in the future surveys (e.g., 24 h). To reduce the analytical error, it is recommended that higher indoor PFABz levels be maintained by using a larger number of PFABz dosers in a room.

### 3.4. Field Study

The 24-h average air exchange rates from outdoors to the dining room & kitchen, Japanese room, and bedroom (mean ± SD, *N* = 6) were 0.2 ± 0.3, 2.4 ± 1.6, and 2.6 ± 2.0 h^−1^, respectively. The 24-h average air exchange rates from the other rooms to the dining room & kitchen, Japanese room, and bedroom (mean ± SD, *N* = 6) were 1.5 ± 0.6, 0.8 ± 0.7, and 2.0 ± 1.2 h^−1^, respectively. The residents kept the windows open for 0–10 h per day. A comparison among the 24-h average air exchange rates for 6 days, revealed that the air exchange rates were high (4.3 and 3.6 h^−1^) when the windows were kept open for 9.5 and 10 hour. This suggested that the human activity pattern (opening of windows) strongly influenced the air exchange rates. There were no windows in the dining room & kitchen. This could be the reason for the low air exchange rates (outdoor to indoor) in the dining room & kitchen.

## 4. Conclusions

In this study, a low-cost and easy-to-use PFT doser with a stable dosing rate was developed to measure the effective air exchange rates in a large-scale survey of air exchange rates, which represents the relationship between the pollutants emitted from indoor sources and the residents’ level of exposure. On the basis of the detection limit and recovery efficiency, it was concluded that HxFBz, OFT, and PFABz can be used as the tracer gases and CS_2_ as the extraction solvent for the measurement of 24-h average air exchange rates. The air exchange rates measured by the proposed modified PFT method were in agreement with these obtained by the CO_2_ concentration decay method.

## Figures and Tables

**Table 1.** Analytical specifications for PFTs.

**Instrument**	**Condition**

GC-MS	Hewlett Packard HP6890-HP5973
Column	Sigma-Aldrich, Inc. Equity-1 capillary column 6 m × 0.32 mm (5 μm)
Carrier Gas	Helium
Flow rate	2 mL min^−1^
Injection volume	1 μL
Injection mode	Splitless
Injection temperature	200 °C
Interface temperature	250 °C
Ion source temperature	230 °C
Column temperature	30 °C (4 min)–(5 °C min^−1^)–180 °C

**Table 2.** Sampling rates, detection limits, recovery rates, and precision.

	LOD [μg mL^−1^]		LOQ [μg mL^−1^]		Recovery efficiency (Mean ± SD)		Sampling rates [mL min^−1^] (Mean ± SD)
	CS_2_ solution	Hexane solution	CS_2_ solution	Hexane solution	CS_2_ extraction	Hexane extraction	
HxFBz	0.0030	0.0029	0.010	0.010	40% ± 3%	<0.29%	10.5 ± 1.1
OFT	0.0036	0.0015	0.012	0.0049	72% ± 5%	18% ± 2%	14.4 ± 1.4
PFABz	0.0045	-	0.015	-	84% ± 6%	-	12.2 ± 0.49
PMCH	0.0098	0.0025	0.033	0.0084	2.7% ± 0.6%	54% ± 18%	-
PDCH	>0.03	0.0031	>0.1	0.010	24% ± 2%	84% ± 21%	-
